# Regional Variation of Pancreatic Cancer Incidence in the Nile Delta Region of Egypt over a Twelve-Year Period

**DOI:** 10.1155/2020/6031708

**Published:** 2020-07-14

**Authors:** Christina Baum, Amr S. Soliman, Heidi E. Brown, Ibrahim A. Seifeldin, Mohamed Ramadan, Breanne Lott, An Nguyen, Ahmed El-Ghawalby, Ahmed Hablas

**Affiliations:** ^1^Department of Epidemiology & Biostatistics, Mel and Enid Zuckerman College of Public Health, University of Arizona, Tucson, AZ 85724, USA; ^2^Department of Community Health and Social Medicine, City University of New York Medical School, New York City, NY 10031, USA; ^3^Gharbiah Cancer Society, Tanta, Gharbiah, Egypt; ^4^Department of Health Promotion Sciences, Mel and Enid Zuckerman College of Public Health, University of Arizona, Tucson, AZ 85724, USA; ^5^Department of Global Health, Milken Institute School of Public Health, George Washington University, Washington, DC 20052, USA; ^6^Department of Surgery, Liver Transplantation Unit, Gastrointestinal Surgery Center, College of Medicine, Mansoura University, Mansoura, Egypt

## Abstract

**Background:**

Pancreatic cancer is one of the deadliest forms of cancer, with incidence rates rising in many countries around the world. Geographic variation in pancreatic cancer incidence has not been studied extensively, especially in low- and middle-income countries. The aim of this study was to characterize the distribution of pancreatic cancer incidence in the central Nile Delta region of Egypt and to examine differences by urban and rural patient residence using the nation's only population-based cancer registry.

**Methods:**

Utilizing the Gharbiah province population-based cancer registry, data were abstracted for 1,089 pancreatic cancer cases diagnosed over twelve years from 1999 to 2010. Age- and sex-specific incidence rates were calculated and compared for urban and rural areas of the eight districts of Gharbiah.

**Results:**

Age-adjusted incidence of pancreatic cancer within Gharbiah varied considerably by urban/rural patient residence and by district. Incidence rates were 1.3 times higher in urban compared to rural areas (4.45 per 100,000 in urban areas and 3.43 per 100,000 in rural areas). The highest incidence rates were observed in urban centers of Kotour, El Santa, and Kafr El-Zayat districts (12.94, 8.32, and 7.89, respectively).

**Conclusion:**

Incidence rates varied greatly by urban and rural areas and by district of residence in the Nile Delta region of Egypt. Future studies should examine potential environmental risk factors that may contribute to the geographic distribution of pancreatic cancer in this region.

## 1. Introduction

Pancreatic cancer is one of the deadliest forms of cancer, causing an estimated 432,000 deaths worldwide each year [1]. Its five-year survival rate of approximately 6% and its propensity for rapid progression make pancreatic cancer a significant global health issue [2]. Most cases are diagnosed in developed regions of the world, as defined by use of the Human Development Index and Gross Domestic Product, accounting for 55% of worldwide incidence [3]. However, both incidence and mortality rates are increasing in less developed countries [3]. Within Northern Africa, age-standardized incidence rates range from 1.0 per 100,000 in Sudan to 4.3 per 100,000 in Libya [4]. Little is known regarding the etiology of pancreatic cancer, but cigarette smoking, diabetes mellitus, exposure to occupational and environmental contamination, alcohol consumption, and genetics have been implicated as risk factors [5–11].

Due to the lethality of pancreatic cancer, mortality rates may act as surrogates for incidence rates. Mortality varies by province in Egypt, with northern districts having an average rate that is 2.85 times the rate of southern districts [12]. This variation may be due to differing environmental exposures, as the northern region of Egypt is also known to have some of the highest levels of soil and water pollution rates in the country [13–15]. A number of studies have identified exposure to environmental pollution, particularly heavy metals, pesticides, and hydrocarbons, as potential risk factors of pancreatic cancer [7, 16–19]. Our previous study included a comparison of the molecular pathology of pancreatic cancer tumors from patients residing in heavily polluted regions of the Nile Delta with the pathology of tumors from patients residing in low-pollution areas and found differences in the types and frequencies of tumor mutations, suggesting the influence of environmental factors [20]. Studying the geographic distribution of pancreatic cancer is essential to gaining a better understanding of how these risk factors may relate to pancreatic cancer etiology. Insights into the spatial distribution of cancer may also guide the geographic allocation of limited cancer prevention and control resources.

While mortality rates of pancreatic cancer across Egypt have been previously reported, this is the first study to describe the geographic distribution of incidence in Egypt. The purpose of this study is to characterize the distribution of pancreatic cancer incidence in the province of Gharbiah using population-based cancer registry data and to lay the groundwork for future etiologic studies to investigate the possible association between regional distribution of pancreatic cancer incidence and potential risk factors.

## 2. Materials and Methods

### 2.1. Study Population and Setting

The study population included all men and women 18 years of age and older who resided in the Gharbiah province and were diagnosed with pancreatic cancer between 1999 and 2010, as recorded in the Gharbiah Population-based Cancer Registry (GPCR) [21]. Data for this study were obtained electronically by abstracting clinical and demographic information of all eligible patients from the registry.

The province of Gharbiah is located in the central Nile Delta region and consists of eight districts with a total population of approximately 4 million people, according to the latest Egyptian census of 2006 [22]. Approximately 72% of the population reside in rural areas, with 28% residing in the eight capital cities of the eight districts [23]. Rural areas tend to be agricultural, while many urban areas in Gharbiah have undergone rapid urbanization and development [24].

### 2.2. Gharbiah Population-Based Cancer Registry (GPCR)

The GPCR, located in the capital city of Tanta, was established in 1998 as a part of the Middle East Cancer Consortium (MECC) and received funding from the U.S. National Cancer Institute in Bethesda and the Egyptian Ministry of Health and Population until being discontinued due to lack of funding in 2011. The GPCR is known to be of high quality in terms of completeness and reliability. The International Association of Cancer Registries (IARC), the Surveillance Epidemiology and End Results (SEER) group of the National Cancer Institute, and the Department of Epidemiology of the University of California at Irvine have conducted validation and control quality checks with the MECC showing high data quality of this registry [25, 26]. The percentage of death certificate only (DCO) cases is comparable between the GPCR (5.5% in men and 4.7% in women), compared to the SEER registries (2.5% and 3.4%, respectively) [4]. The data from the GPCR have been included in two volumes of the international cancer publication, Cancer Incidence in Five Continents, and the GPCR is Egypt's only population-based cancer registry [4, 25].

The GPCR used active case finding to collect information on all cancer cases within the entire Gharbiah province. Pancreatic cancer cases included in the registry were classified based on the World Health Organization's ICD-O-2 coding for 1999-2000, followed by the ICD-O-3 coding from 2001 onward [27]. The following codes were included, representing all cases in the registry: C25.0, C25.1, C25.2, C25.3, C25.4, C25.7, C25.8, and C25.9. This includes all subtypes of pancreatic cancer. Information abstracted from the registry included case registry number, age at diagnosis, sex, district, urban/rural residence, basis of diagnosis, smoking status, occupation, and referral hospital. All personal identifiers were removed from the analyzed data to protect patient confidentiality. The study was approved by the University of Arizona's Institutional Review Board and the Gharbiah Cancer Society's Ethics Committee.

### 2.3. Census Data

Population data for each district of Gharbiah were obtained from the 1996 and 2006 censuses conducted by the Central Agency for Public Mobilization and Statistics (CAPMAS) [22]. The census consisted of 16 age categories of five-year age intervals for each district. It classified residence into two distinct categories; those who lived within the capital city of each district were considered urban, where the remaining towns and villages were considered rural. Each patient was assigned a residence code based on their residential address that aligned with CAMPAS coding to classify urban or rural cases. Using the total population of Gharbiah from the 1996 and 2006 census data, an annual growth rate (AGR) was calculated using the following formula: AGR = ((2006 pop − 1996 pop)/1996 pop)/10. This growth rate (1.9%) was then used to estimate the yearly populations of each district, except for the district of Kotour. For Kotour, we observed a distinctly higher annual growth rate of 3.8%, so we utilized this specific rate for Kotour only to ensure accurate population estimates. The population estimates formed the denominators in calculating age- and sex-specific incidence rates.

### 2.4. Statistical Analysis

Data were obtained electronically from the GPCR, and descriptive statistics were generated using SAS Studio 3.8. Crude age- and sex-specific incidence rates (IR) were then calculated by dividing the number of pancreatic cancer cases per group (E) by the corresponding age- and sex-specific population estimates calculated with CAPMAS census data. Age-standardized rates were then calculated by direct standardization utilizing the Segi-Doll world standard [21]. Incidence rate ratios (IRRs) were also computed to compare urban and rural incidence rates of each district. The confidence intervals (CI) of the IRRs were calculated using the following formula: CI = exp [In(IR1/IR2)] ± 1.96 × √(1/E1 + 1/E2).

Shapefiles for Egyptian provinces and districts were downloaded from the Humanitarian Data Exchange and imported into ArcMap 10.6.1. A map layer created by the team was utilized to discriminate between urban and rural areas. Incidence data were imported into ArcMap in the form of a geodatabase, and choropleth maps were created.

## 3. Results

A total of 1,089 pancreatic cancer cases (36.3% female and 63.7% male) were reported to the GPCR from 1999 to 2010 and included in this study (Table 1). Most of the study subjects resided in Tanta (26.2%), Mehalla (26.8%), and Kafr El-Zayat (10.8%) districts, with 59.7% of cases residing in rural areas. The majority of the cases were diagnosed at the Tanta Cancer Center (50.5%) and the Mansoura Gastroenterology Surgical Center (24.7%). A total of 398 cases (36.5%) were diagnosed using microscopic verification, including histology of either the primary tumor or metastasis, or cytology. Approximately half of the cases were younger than 60 years old (50.9%). Information on smoking and occupation was not available for all patients (31.6% missing values for smoking and 82.9% missing values for occupation) and therefore is excluded from Table 1.

**Figure 1:**
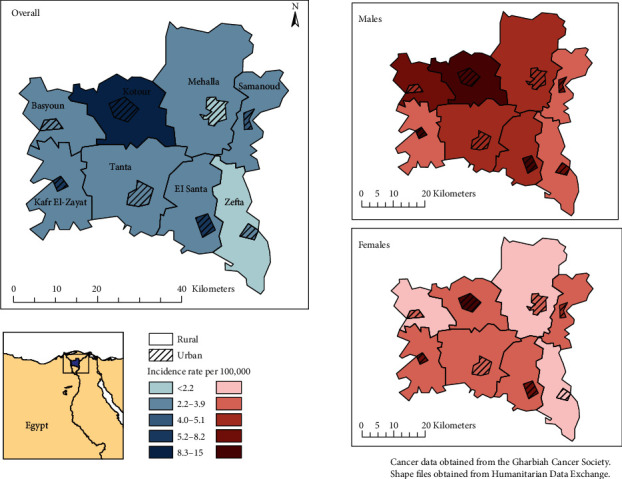
Map of pancreatic cancer incidence rates in urban and rural Gharbiah for males and females (1999-2010).

**Figure 2:**
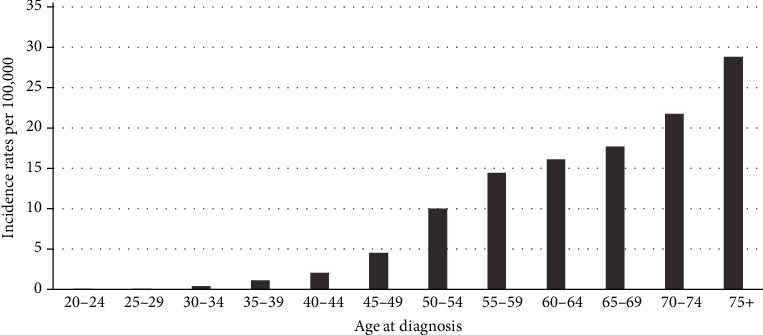
Age-specific pancreatic cancer incidence rates per 1,089 cases in the Gharbiah district of Egypt (1999-2010).

Figure 1 and Table 2 present the age-adjusted incidence rates for urban and rural areas in all eight districts of Gharbiah, stratified by sex. Incidence rates varied by district, ranging from 2.46 per 100,000 in Zefta (southeastern Gharbiah) to 6.91 per 100,000 in Kotour (northwest Gharbiah). The districts with the highest overall incidence rates were Kotour, Kafr El-Zayat, and Mehalla (6.91, 4.18, and 3.93 per 100,000, respectively).  Figure 2 also displays the age-specific incidence rates, highlighting the correlation between pancreatic cancer and increasing age. As has been reported across the globe [2], our study shows that pancreatic cancer predominantly affects the older population, with 80.3% of cases diagnosed at age 50 years or above.

In all districts, incidence rates were higher in urban areas (4.45/100,000) compared to rural areas (3.43/100,000). The highest incidence rates were observed in urban centers of Kotour, El Santa, and Kafr El-Zayat districts (12.94, 8.32, and 7.89 per 100,000, respectively). The districts with the highest urban-rural incidence rate ratios (IRRs) include El Santa (IRR = 2.48, 95%CI = 2.18‐2.81), Kafr El-Zayat (IRR = 2.46, 95%CI = 2.29‐2.64), and Kotour (IRR = 2.01, 95%CI = 1.57‐2.41). The province-level (Gharbiah) age-standardized rate was 3.78 per 100,000 (4.87 and 2.72 per 100,000 for men and women, respectively) (Table 2).

## 4. Discussion

This was the first study to investigate the geographic distribution of pancreatic cancer incidence in Egypt. The study revealed two interesting trends. First, the results showed that incidence rates were significantly higher in urban compared to rural districts. Second, the incidence rates of pancreatic cancer varied greatly by district.

Regarding the difference between urban and rural areas, we found that urban areas had incidence rates that were 1.3 times the rates of rural areas (95% CI: 1.29-1.31). The highest urban incidence was found in Kotour (14.28 per 100,000 in males and 11.27 per 100,000 in females) compared with the lowest rural incidence rate in Zefta (2.50 per 100,000 in males and 1.87 per 100,000 in females). These findings are in concordance with two studies conducted in China which demonstrated that pancreatic cancer incidence and mortality rates are higher in urban compared to rural areas [28, 29]. Another study which estimated pancreatic cancer incidence using hospitalization data in Brazil also found that estimated incidence rates were higher in urban compared to rural regions [30]. These findings were hypothesized to be related to the process of urbanization and the risk factors that accompany socioeconomic development such as increased smoking rates, adoption of westernized diets, lack of exercise, increased diabetes rates, changes in the urban environment, and improvement in diagnosis [28–30]. One study in Japan, however, found that there were no significant differences in pancreatic cancer mortality between metropolises, cities, and counties but offered no explanation for these results, which may have differed from other studies due to the manner in which municipalities were classified [31].

We believe it is unlikely that the observed differences between urban and rural rates were due to disparities in access to medical care. Across Egypt, 95% of the population lives within 5 km of a primary health care facility [32]. Within Gharbiah, a large number of health care facilities in addition to prolific and inexpensive transportation options facilitate accessibility of medical services. The most distant location in Gharbiah is less than 50 km from either of the top two pancreatic cancer diagnosing clinics (Tanta Cancer Center and Mansoura Gastroenterology Center), with smaller health centers in the capital cities less than 20 km away from the most rural areas. The Ministry of Health and Population also provides free health care to all citizens, minimizing health care cost as a factor influencing accessibility [33]. These factors give us assurance that urban and rural differences are not related to accessibility and affordability of health care.

Differences in urban and rural lifestyles and occupational exposures are likely to contribute to the observed differences in urban and rural incidence rates. Obesity and diabetes have been shown to vary significantly between urban and rural parts of Egypt; according to a 1995 study, 49% of urban populations with higher incomes in Egypt were obese and 20% had diabetes, compared to rural populations where only 16% were obese and 4.9% had diabetes [34]. These figures have continued to climb since then, with an estimated 63.5% of Egyptian adults overweight or obese in 2016 and 15.6% overall diabetes prevalence in 2011 [35, 36]. A study in southern Egypt which examined dietary factors in relation to pancreatic cancer risk also showed that a higher caloric intake was associated with pancreatic cancer [37]. As urban residents have greater exposure to Westernized diets, characterized in part by energy-dense foods, dietary pattern differences between urban and rural households could be potentially contributing to elevated incidence for urban residents. While there may be differences in cigarette smoking habits between urban and rural populations, the prevalence of smoking in rural areas has not been well documented and lack of smoking status for cases in this study prevented analysis of any potential association [38]. In 2002, 35% of Egyptian men and 1.6% of Egyptian women were estimated to be smokers, which may contribute to the differences in male and female incidence rates [39]. More recently, the World Health Organization estimated that 46.4% of men and 0.2% of women in Egypt were current cigarette smokers [40]. Other types of tobacco use such as waterpipes or “hookah” may also be important to investigate. One study found that rural Egyptian men perceived waterpipe use to be less harmful than cigarette smoking and were also less likely to contemplate quitting or avoiding tobacco use [41]. As smoking is one of the strongest risk factors for pancreatic cancer [2], further research would benefit from improved documentation of urban versus rural smoking and tobacco-use behavior.

In regard to occupational exposures, a hospital-based study conducted in the nearby province of Dakahleia found that farming, which is highly correlated with rural residence, was significantly associated with pancreatic cancer risk [42]. Our study, however, found that rural, predominantly agricultural areas, exhibited lower incidence rates. Another hospital-based study showed that pancreatic cancer cases who were both farmers and who reported a history of handling pesticides were 2.6 times more likely to have pancreatic cancer compared with farmers reporting no handling history [19]. It is possible, then, that urban occupational exposures are equally important as rural agricultural exposures or that the pattern of urban versus rural incidence rates which we observed was due to even more influential risk factors affecting urban populations. Other types of occupations occurring in urban areas with industrial exposure to pesticides and carcinogenic materials such as manufacturing, soldering, and metalworking have not been studied specifically in relation to pancreatic cancer in Egypt.

Regarding the observed regional variation, our results are consistent with other epidemiological studies demonstrating regional variation of pancreatic cancer incidence and mortality rates within the U.S., China, Japan, and France [12, 28, 43–45]. Our results are also in agreement with studies showing that pancreatic cancer incidence rates are lower in developing nations compared to northern, more developed countries [3, 12, 46]. The age-standardized rate of Gharbiah calculated in this study is 3.78 per 100,000, compared to 7.7 per 100,000 in the United States [1].

As the districts in Gharbiah are relatively homogenous in terms of lifestyle, age distributions, and sex ratios, regional variation in incidence rates may be attributed to differences in exposure to environmental risk factors. It is well documented that the Nile Delta region has some of the highest levels of environmental contamination in Egypt by heavy metals, hydrocarbons, and pesticides; pollution levels are particularly high in urban and industrial areas [13, 47]. Cadmium, a known carcinogen and heavy metal, has also shown to be associated with pancreatic cancer cases and may contribute to the variation in incidence by district [42, 48].

This study has multiple strengths. Mainly, it is the first study to characterize the geographic distribution of pancreatic cancer incidence in Egypt. The GPCR is a high-quality registry that received regular quality checks by the IARC and is routinely cited by Cancer Incidence in Five Continents [4, 25]. The study also utilized a large sample size for this rare cancer spanning twelve years. The population of Gharbiah is stable; people are likely to live in the same village or city for the entirety of their lives, and the migration rate throughout this period remained low [49]. Furthermore, 35% of the cases used in this study were diagnosed by histopathology, a proportion comparable to other studies on pancreatic cancer from Western countries [50].

With these strengths, the study had limitations. There is the possibility that in Egypt and in other regions of the world, people may die of pancreatic cancer before seeking medical care or without obtaining a medical diagnosis first. However, we have no reason to believe that this would lead to differential misclassification. Because population-based cancer registries are not designed for specific research investigations, the variables in the registry were limited to clinical and descriptive epidemiologic information. Well-designed case-control studies that can assess individual risk factors such as smoking frequency, occupational exposures, and diet are needed. Additionally, although we had a reasonable sample size of 1,089 patients for this rare cancer, we were unable to conduct analyses on a finer spatial resolution. Utilizing more nuanced urbanization classifications, such as distinguishing semiurban areas, would also be useful to examine whether there is a linear correlation between pancreatic cancer incidence and the rate of urbanization. The population estimates used to calculate incidence differed slightly from the years that cases were reported but were utilized based on the availability of census data from 1996 and 2006. Lastly, while we are able to provide rationale for the differential rates by urbanization, data were not available to definitively establish causality.

## 5. Conclusions

This study demonstrates that pancreatic cancer incidence is higher in urban compared to rural areas in Gharbiah, Egypt. The study also reveals geographic variation in pancreatic cancer incidence by district of residence. It highlights the importance of cancer registration in Egypt and a need for future registration to provide relevant public health insights. This study provides a preliminary examination into pancreatic cancer distribution in Egypt; however, we recommend the completion of more advanced geospatial analyses with larger datasets to confirm these initial findings. In a country undergoing great urban growth, understanding the epidemiology of pancreatic cancer and investigating the risk factors which may vary between urban and rural settings and between districts will be essential for gaining a better understanding of the disease etiology and for guiding cancer prevention and control strategies.

## Figures and Tables

**Table 1:** Clinical and demographic characteristics of 1,089 pancreatic cancer cases in Gharbiah during the period of 1999-2010 by urban and rural places of residence.

Variable	Description	Urban no. (%)	Rural no. (%)	Total cases
Year of diagnosis	1999	36 (8.20)	31 (4.77)	67 (6.15)
	2000	30 (6.83)	38 (5.85)	68 (6.24)
	2001	29 (6.61)	35 (5.38)	64 (5.88)
	2002	41 (9.34)	42 (6.46)	83 (7.62)
	2003	36 (8.20)	45 (6.92)	81 (7.44)
	2004	44 (10.02)	44 (6.77)	88 (8.08)
	2005	29 (6.61)	62 (9.54)	91 (8.36)
	2006	42 (9.57)	58 (8.92)	100 (9.18)
	2007	39 (8.88)	65 (10.00)	104 (9.56)
	2008	31 (7.06)	77 (11.85)	108 (9.92)
	2009	44 (10.02)	83 (12.77)	127 (11.66)
	2010	38 (8.66)	70 (10.77)	108 (9.92)
	All years	439	650	1089

Age	0-24	1 (0.23)	3 (0.46)	4 (0.37)
	25-29	0 (0.00)	3 (0.46)	3 (0.28)
	30-34	7 (1.59)	8 (1.23)	15 (1.38)
	35-39	13 (2.96)	23 (3.54)	36 (3.31)
	40-44	17 (3.87)	38 (5.85)	55 (5.05)
	45-49	37 (8.43)	65 (10.00)	102 (9.37)
	50-54	73 (16.63)	90 (13.85)	163 (14.97)
	55-59	71 (16.17)	105 (16.15)	176 (16.16)
	60-64	76 (17.31)	103 (15.85)	179 (16.44)
	65-69	52 (11.85)	84 (12.92)	136 (12.49)
	70-74	46 (10.48)	66 (10.15)	112 (10.28)
	75+	46 (10.48)	62 (9.54)	108 (9.92)

Sex	Female	273 (62.19)	421 (64.77)	694 (63.73)
	Male	166 (37.81)	229 (35.23)	395 (36.27)

Basis of diagnosis^1^	Death certificate only	54 (12.41)	19 (2.92)	73 (6.73)
	CT scan/US/MRI	60 (13.79)	128 (19.69)	188 (17.33)
	Exploratory surgery/ERCP	103 (23.68)	158 (24.31)	261 (24.06)
	Radiology with tumor marker	60 (13.79)	105 (16.15)	165 (15.21)
	Cytology/hematology	9 (2.07)	12 (1.85)	21 (1.94)
	Histology of metastases	32 (7.36)	87 (13.38)	119 (10.96)
	Histology of primary	116 (26.7)	141 (21.69)	257 (23.69)
	Autopsy with histology	1 (0.23)	0 (0.00)	1 (0.09)

District	Tanta	148 (33.71)	137 (21.08)	285 (26.17)
	Mehalla	153 (34.85)	139 (21.38)	292 (26.81)
	Kafr El-Zayat	46 (10.48)	72 (11.08)	118 (10.84)
	Zefta	24 (5.47)	55 (8.46)	79 (7.25)
	Samanoud	21 (4.78)	51 (7.85)	72 (6.61)
	El Santa	19 (4.33)	81 (12.46)	100 (9.18)
	Kotour	13 (2.96)	65 (10.00)	78 (7.16)
	Basyoun	15 (3.42)	50 (7.69)	65 (5.97)

^1^Four cases (0.37%) had missing data on the basis of diagnosis.

**Table 2:** Age-adjusted pancreatic cancer incidence rates per 100,000 with 95% confidence intervals for 1,089 cases in Gharbiah by district, sex, and urban/rural place of residence (1999-2010).

Region	Urban				Rural				Total			
	M	F	Overall	No. cases	M	F	Overall	No. cases	M	F	Overall	Number of cases
Tanta	4.45	3.59	4.04	148	4.77	2.76	3.70	137	4.60	3.16	3.88	285
	(3.52-5.39)	(2.69-4.49)	(3.39-4.70)		(3.74-5.79)	(2.03-3.50)	(3.08-4.32)		(3.91-5.29)	(2.85-3.73)	(3.43-4.33)	

Mehalla	4.63	3.34	4.05	153	5.65	2.12	3.84	139	5.08	2.71	3.93	292
	(3.68-5.57)	(2.50-4.17)	(3.41-4.69)		(4.54-6.76)	(1.46-2.79)	(3.20-4.48)		(4.37-5.80)	(2.18-3.25)	(3.48-4.38)	

Kafr El-Zayat	9.58	5.93	7.89	46	4.02	2.48	3.21	72	5.31	3.08	4.18	118
	(6.21-12.95)	(2.93-8.93)	(5.61-10.17)		(2.82-5.22)	(1.57-3.38)	(2.47-3.95)		(4.10-6.53)	(2.17-3.99)	(3.42-4.93)	

Zefta	5.58	1.42	3.58	24	2.50	1.87	2.18	55	3.19	1.76	2.46	79
	(3.13-8.02)	(0.03-2.81)	(2.15-5.01)		(1.62-3.37)	(1.12-2.62)	(1.60-2.75)		(2.32-4.07)	(1.11-2.41)	(1.92-3.01)	

Samanoud	5.77	4.59	5.15	21	3.61	2.39	2.96	51	4.08	2.71	3.38	72
	(2.63-8.91)	(1.41-7.76)	(2.95-7.36)		(2.32-4.90)	(1.37-3.41)	(2.15-3.77)		(2.86-5.30)	(1.72-3.70)	(2.60-4.16)	

El Santa	11.02	5.44	8.32	19	4.69	2.22	3.36	81	5.24	2.45	3.76	100
	(5.03-17.01)	(1.09-9.80)	(4.58-12.06)		(3.41-5.96)	(1.41-3.02)	(2.63-4.09)		(3.97-6.52)	(1.64-3.26)	(3.02-4.50)	

Kotour	14.28	11.27	12.94	13	10.42	3.06	6.45	65	10.70	3.62	6.91	78
	(3.70-24.85)	(2.25-20.29)	(5.91-19.98)		(7.44-13.40)	(1.65-4.48)	(4.88-8.01)		(7.84-13.55)	(2.17-5.07)	(5.38-8.44)	

Basyoun	4.40	2.38	3.43	15	5.29	2.06	3.57	50	5.09	2.15	3.56	65
	(1.67-7.13)	(0.29-4.46)	(1.69-5.16)		(3.54-7.05)	(1.02-3.10)	(2.58-4.56)		(3.60-6.58)	(1.21-3.09)	(2.69-4.42)	

Total	5.21	3.63	4.45	439	4.68	2.31	3.43	650	4.87	2.72	3.78	1089
	(4.59-5.82)	(3.08-4.19)	(4.04-4.87)		(4.23-5.12)	(2.01-2.61)	(3.17-3.70)		(4.51-5.23)	(2.46-2.99)	(3.56-4.01)	

## Data Availability

All data used in the study were provided by the Gharbiah Cancer Society. Data are made available to researchers following a formal proposal for a specific study and request for data.
